# Cutting Parameter Selection for Efficient and Sustainable Repair of Holes Made in Hybrid Mg–Ti–Mg Component Stacks by Dry Drilling Operations

**DOI:** 10.3390/ma11081369

**Published:** 2018-08-07

**Authors:** Eva María Rubio, María Villeta, José Luis Valencia, José Manuel Sáenz de Pipaón

**Affiliations:** 1 Department of Manufacturing Engineering, Industrial Engineering School, Universidad Nacional de Educación a Distancia (UNED), St/Juan del Rosal 12, E28040 Madrid, Spain; jm@saenzdepipaon.com; 2 Department of Statistics and Data Science, Faculty of Statistical Studies, Complutense University of Madrid (UCM), Ave. Puerta de Hierro 1, E28040 Madrid, Spain; mvilleta@estad.ucm.es (M.V.); joseval@estad.ucm.es (J.L.V.)

**Keywords:** hybrid components stack, titanium, magnesium, repair and maintenance operations, drilling, dry machining, roughness average (*Ra*), ANOVA

## Abstract

Drilling is one of the most common machining operations in the aeronautic and aerospace industries. For assembling parts, a large number of holes are usually drilled into the parts so that they can be joined later by rivets. As these holes are subjected to fatigue cycles, they have to be checked regularly for maintenance or repair, since small cracks or damage in its contour can quickly cause breakage of the part, which can have dangerous consequences. This paper focuses on finding the best combinations of cutting parameters to perform repair and maintenance operations of holes in stacked hybrid magnesium–titanium–magnesium components in an efficient, timely, and sustainable (without lubricants or coolants) manner, under dry drilling conditions. For the machining trials, experiments were designed and completed. A product of a full factorial 2^3^ and a block of two factors (3 × 2) was used with surface roughness as the response variable measured as the mean roughness average*.* Analysis of variance (ANOVA) was used to examine the results. A set of optimized tool and cutting conditions is presented for performing dry drilling repair operations.

## 1. Introduction

Reduction in energy consumption is a constant demand in various industries, such as aeronautic, aerospace, and automotive, due to economic and environmental reasons. However, as energy consumption is closely linked to weight, one method to reduce energy consumption is to use lighter components composed of light alloys, such as titanium (Ti), aluminium (Al), and/or magnesium (Mg), all of which have an excellent weight/mechanical properties ratio.

The parts used in the aforementioned industries have to be rigid, strong, and light. In addition, they have to meet high precision and quality standards. As such, the production of these light alloys has been extensively studied [1,2,3,4,5,6,7,8,9,10,11,12,13,14,15,16,17,18,19,20,21,22,23,24,25,26,27,28,29,30,31,32,33]. Their repair and maintenance has also been thoroughly studied. Given their uniqueness and high cost, replacement parts are often not available when they have to be maintained or repaired [34,35,36,37,38,39,40,41,42,43,44,45,46,47,48,49].

The development of new alloys or polymers that increase the number of applications may be expensive but not always achievable. Sometimes, even when a material exists, it struggles to simultaneously satisfy all the necessary properties for concrete application using the material alone. Then, the combination of two or more materials is an alternative for producing hybrid components whose properties satisfy the requirements in areas not covered by the individual components separately [50,51,52,53,54,55,56,57,58]. Different combinations of materials and technologies have been studied in order to improve their properties [50,51,52,53,54,55,56,57,58,59,60,61,62,63,64,65,66,67,68,69,70], so the use of hybrid structures is becoming more common.

The complexity of aeronautic and aerospace parts means that most have to be mechanized until achieving their final geometry. Then, using hybrid components, machining together several materials simultaneously is necessary. Drilling is one of the most usual machining operations in these industrial sectors, since it is used to create a large number of holes for assembling the parts. As these holes are subjected to fatigue cycles, every so often they must be checked for their maintenance since small cracks or damage in its contour can quickly degenerate into the breakage of the piece. Given the nature of these environments, breakages can have serious consequences. Due to the cost and complexity of these types of pieces, replacement parts are not maintained in stock to be used for repair or maintenance [3,36,49].

Performing machining operations in complex geometries of hybrid components or stacks is a significant challenge in terms of accuracy and quality, especially when the combined materials are magnesium and titanium. Magnesium is the lighter metal and has good mechanical properties that make magnesium alloys attractive for applications in industries where weight is crucial. Titanium is in demand due to its physical, chemical, and thermal properties. However, both materials present some problems. Magnesium is easily ignited by the heat generated during the machining process. The low thermal conductivity of titanium increases risk of tool wear, its low modulus means that parts move away from the cutting tool during machining, and its high chemical reactivity and low hardness tend to produce cracks in the cutting tools [71,72,73].

For solving these problems, different compatible and environmentally sustainable lubricants and coolants have been developed [26,38,39,40,45,74]. However, more research is needed because machining hybrid components causes an increase in the instability of the process due to the different cutting characteristics of the different materials. Many investigations have been reported for the simultaneous machining of materials in the search for optimal combinations of cutting conditions, tools, and cooling systems [56,57,75,76,77,78,79,80,81,82,83,84,85,86,87,88,89].

This paper focuses on drilling processes, and especially on finding the best combinations of cutting conditions and type of tools to perform hole repair and maintenance operations with dry machining on hybrid stacks of Mg–Ti–Mg in the shortest time and most sustainable method possible.

**Figure 1:**
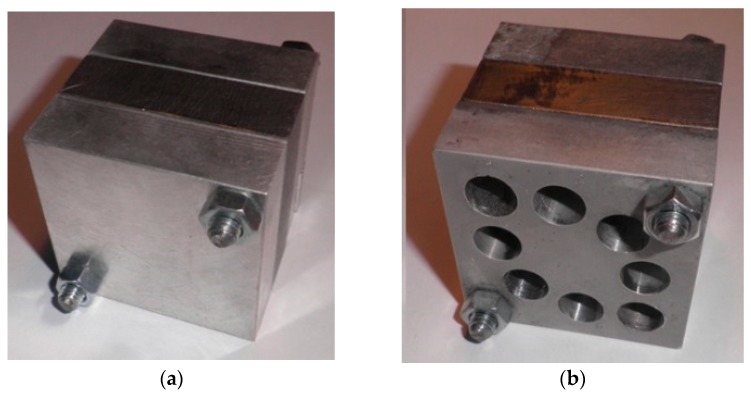
Hybrid magnesium–titanium–magnesium stack for (**a**) drilling and (**b**) hole repair trials [56,57].

The magnesium–titanium–magnesium stack (Figure 1) was predrilled to simulate repair operations in hybrid components that are assembled with rivets. The two parts of magnesium were considered the base of the stack and the titanium was considered the insert.

When a rivet hole is damaged, it has to be drilled to a larger diameter and an oversized rivet has to be assembled. This repair process is widely used in aeronautics and has to be performed with care as cracks in the structure can produce catastrophic consequences.

In this work, the surface roughness on the inside the holes was obtained by drilling the hybrid stacks. The stacks were composed of a magnesium alloy and titanium alloy to determine if it is possible to efficiently and sustainably repair or maintain aeronautic and aerospace industry parts with very strict surface finish requirements. To achieve this goal, a design of experiments (DOE) was defined for the drilling trials and the surface roughness measurements. The design selected was the product of a full factorial 2^3^ and a block of two factors (3 × 2), whose possible influential cutting parameter factors were feed rate, *f*, cutting speed, *V*, and tool coating type, *T*. The location factors in terms of measuring the surface roughness included location on the specimen, *LRS*, and location on the insert, *LRI*. The obtained results were analyzed via analysis of variance (ANOVA), which helped establish a ranking of the parameter combinations based on the surface roughness achieved in the machining trials and helped determine the optimum combination of factors for performing these types of operations.

## 2. Materials and Methods

We followed the guidelines provided by Montgomery [87]. First, in the planning stage, factors, levels, ranges, and response variables were determined (Table 1). Then, the design of experiments (DOE) was performed. The design was elected according to the fixed resources and objectives. In this case, the goal was to analyze the variability in the surface roughness inside the holes obtained by drilling of hybrid stacks composed of UNS M11917 magnesium alloy and UNS R56400 titanium alloy (Table 2). The design selected was the product of a full factorial 2^3^ and a block of two factors (3 × 2; *LRI* × *LRS*) with a total of 48 experimental runs, as can be observed in Table 2. In this table, each line represents two experimental runs, one for each level of the *LRI* factor.

Next, the machining trials were completed. To execute the drilling trials, it was first necessary to establish the protocols both for the cutting conditions to be used and for registering the obtained data. Then, it was necessary to prepare the workpieces with the specimens of the hybrid stacks, the tools, and the machine tool, introducing the cutting conditions selected. Next, the machining operations were performed, and finally, we photographed and recorded videos of all the trials for further analysis once the process was finished.

Then, we measured the response variable. Surface roughness was selected as the response variable, measured as the roughness average (*Ra*) [88]. Once the data were obtained, we performed a statistical analysis. The variability of the average roughness values, *Ra*, was modelled using ANOVA, identifying both the influential factors and interactions among them on surface roughness. In addition, an exploratory data analysis was performed to obtain a clear graphical view of the key aspects in terms of the distribution of the influential factors on the surface finish of hybrid magnesium–titanium–magnesium stacks. The relationships between pairs of influential factors were illustrated and analyzed. After the statistical analysis of the results, some conclusions were established.

## 3. Trials

This work focused on the manufacturing process of drilling and on repair operations of holes used to join parts of different materials by means of rivets. Materials, cutting tools, cutting conditions, and measurement locations were established as influential factors on the variability in surface roughness.

### 3.1. Specimens: Materials and Geometries

**Figure 2:**
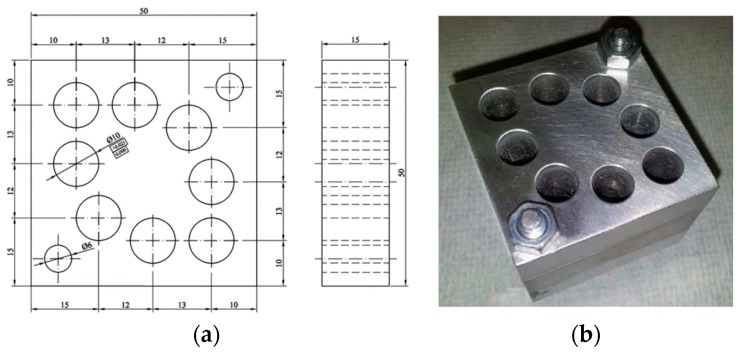
(**a**) Design of the geometry of each of the three parts of the workpiece; (**b**) Predrilled workpiece used during the trials to simulate hole repair operations on hybrid stacks [56,57].

The materials used in the manufacture of the workpieces for hole repair drilling operations included magnesium alloy UNS M11917 and titanium alloy UNS R56400, whose compositions are outlined in Table 3. In the absence of standards, national or international, in relation to the design and manufacture of test pieces, we decided to use 50 × 50 × 15 mm parallelepipeds (Figure 2) [57]. The hybrid component stacks were composed of a total of three pieces or parallelepipeds called specimens: two of magnesium alloy and one of titanium alloy. The two UNS M11917 magnesium alloy specimens were called the base and the UNS R56400 titanium alloy specimen, located between the two magnesium pieces, was called the insert; this is similar to previous works involving other processes and other materials analyzed. The specimens were positioned one above the other to differentiate the measurements when collecting data. The three parallelepipeds or specimens were mechanically fixed together so that it was possible to disassemble and measure the surface roughness inside the machined holes with relative ease. In order to simulate repair and maintenance operations, a test piece was predrilled with a number of holes in accordance with the requirements for the planned experiments.

### 3.2. Tools

**Figure 3:**
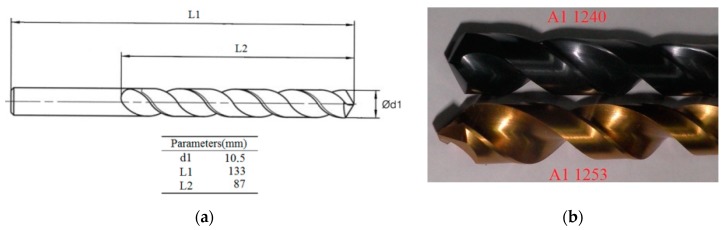
(**a**) Drill tool geometry and dimensions; (**b**) Materials and uses of the tools: A11240 is composed of High-Speed Steel and recommended for titanium, steel, and stainless steel and A11253 is composed of High-Speed Steel coated with titanium nitride and recommended for steel, stainless steel, titanium, aluminium alloys, and copper alloys [64].

Two types of tools were selected for drilling operations with the same geometry (Figure 3a) but with different coatings (Figure 3b) (GARANT, Hoffmann Iberia Quality Tools S.L., San Fernando de Henares, Madrid, Spain). We selected tools made of High-Speed Steel (HSS CO): two-flute twist drills with 130° point angle and in two different qualities, A11240 and A11253. A11240 is recommended for titanium, steel, and stainless steel, whereas A11253 is coated with titanium nitride (TiN) and recommended for steel, stainless steel, titanium, aluminium alloys, and copper alloys. This selection allows us to use the tools in the largest number of trials involving different material combinations, considering that this work is inside a wider project that involves different machining processes (turning, milling, drilling), materials (steel, aluminium, titanium, and magnesium), tools, and workpieces of several types, shapes, and sizes.

### 3.3. Cutting Parameters

**Figure 4:**
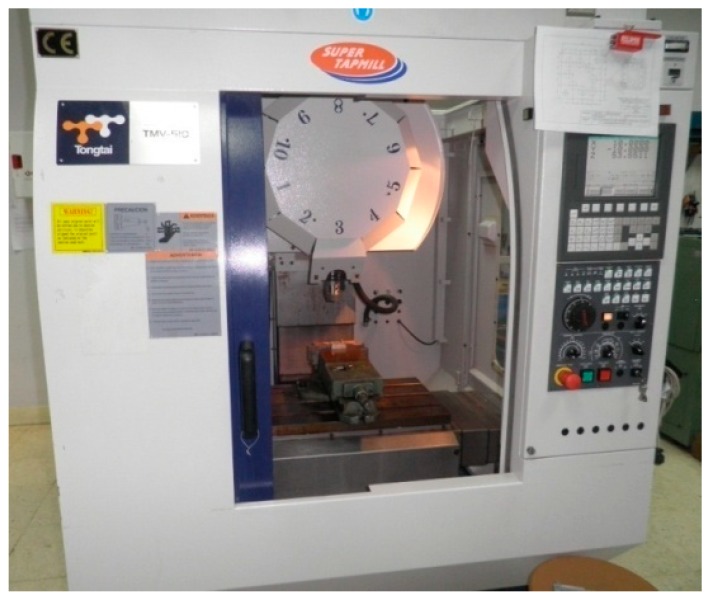
Tongtai TMV510 machining center equipped with a Fanuc Control Numeric Computer (CNC).

The trials were carried out in a Tongtai TMV510 machining center (Tongtai Machine & Tool Co., Luzhu Dist, Kaohsiung City, Taiwan) equipped with a Fanuc Control Numeric Computer (CNC) (FANUC Iberia, Castelldefels, Barcelona, Spain) (Figure 4) under dry conditions. The values usually used during repair and maintenance operations were selected for the factor levels of feed rate, *f*: 50 mm/min and 100 mm/min; cutting speed, *V*: 20 m/min and 25 m/min; and depth of cut, *d*: 0.25 mm.

### 3.4. Measurement Locations

**Figure 5:**
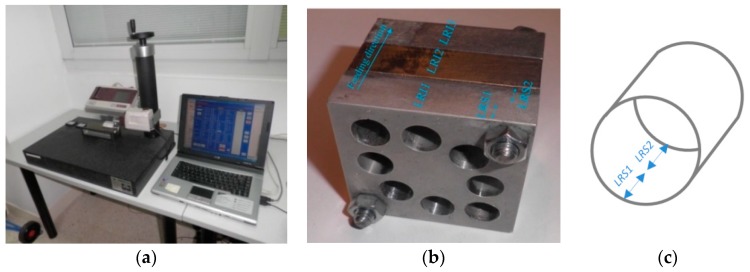
(**a**) Mitutoyo Surftest SJ 401 roughness tester; (**b**) location of the measurements of *LRS* and *LRI* along the feeding direction, inside each hole; and (**c**) detail of the specific measurement locations in the holes of each specimen.

The measurements of the surface roughness were recorded using a Mitutoyo Surftest SJ 401 roughness tester (Mitutoyo America Corporation, Aurora, IL, USA) (Figure 5a). From Table 1, the measurement locations along with the specimen and the insert were used as influential factors (Figure 5b). These factors are *LRS* for the specimen and *LRI* for the insert. For the *LRS*, two values were chosen: *LRS1*, the roughness at the beginning of the hole, and *LRS2*, the roughness at the end of the hole along the feeding direction. Specifically, *LRS1* was recorded within the first 7 mm of the hole and *LRS2* was recorded within the last 7 mm of the hole (Figure 5c). For the *LRI*, three levels were established according to the drilling direction: *LRI1*, the roughness before the insert (first specimen), which is the roughness inside the holes of the first magnesium piece; *LRI2*, the roughness on the insert (second specimen), which is the roughness inside the holes of the titanium piece; and *LRI3*, the roughness after the insert, which is the roughness inside the holes of the second magnesium piece (third specimen).

### 3.5. Factors and Levels Selected

The values selected for the levels of factors analyzed in this study are shown in Table 4. The depth of cut, *d*, was recorded at 25 mm and was equal in all tests. This value was selected to simulate repair and maintenance operations in which it is necessary to adhere to the tolerance dimensions established during the design of the parts.

## 4. Results, Analysis, and Discussion

### 4.1. Results

The surface roughness in terms of the roughness average, *Ra*, was measured after performing all the drilling trials inside all the holes and in the different locations established in Table 4, following the direction and locations shown in Figure 5b,c for each hole. The results obtained for the 48 *Ra* experimental values are outlined in the last two columns of Table 5.

We firstly assessed the initial obtained results to determine any trend and to compare the findings with those obtained in previous works in which the materials were studied separately or along with other materials. The *Ra* mean values in the three measured zones are provided in Table 6.

The values obtained in this work aligned with those in other previous works about titanium using a similar feed rate, cutting speed, or point angle [81,89,90,91], as well as with those obtained when drilling magnesium matrix composites [83]. For optimizing magnesium alloys during drilling operations, other point angles are suitable; however, similar surface roughness values were obtained in this work [92].

### 4.2. Analysis and Discussion

In order to statistically analyze the experimental *Ra* data collected (Table 5), a fixed effects ANOVA was performed to examine the interactions up to second order and excluding an effect each time. The selection criteria of the significant effects in the ANOVA after each iteration were as follows [93]: in each new ANOVA, the effect with a higher *p*-value (which was therefore less statistically significant) was excluded; the backward algorithm finishes when all the effects that remain in the ANOVA have a *p*-value lower than 0.05.

The final outcome of this iterative ANOVA algorithm over the experimental *Ra* data did not lead to any conclusive result. For that reason, a logarithmic transformation of the experimental *Ra* data was performed. Such a transformation allows maintaining the order of the original *Ra* data while smoothing the impact of the outliers. The outcome of the first iteration of the ANOVA over the transformed *Ra* data—over the *Ra* Naperian logarithm ln*Ra*—is provided in Table 7. A second iteration for the ANOVA was then completed for the effects contained in the table, excluding the LRI*V effect which had a maximum *p*-value of 0.828.

The final result of the backward algorithm for the last iteration is displayed in Table 8. In this table, all the *p*-values are lower than 0.05, so the three effects in the first column of this table can be considered statistically significant. Therefore, as a consequence of the ANOVA, we concluded that the interaction between type of tool and the location with respect to the insert T*LRI, the location with respect to the insert *LRI*, and the interaction between type of tool and feed rate T*f are the three effects among the 15 analyzed with a significant statistical influence on the surface finish of the machining on the dry drilling stack, composed of magnesium alloy UNS M11917 and titanium alloy UNS R56400. For example, the *LRS* effect, which measures the roughness differences between the beginning and end of the specimen along the feeding direction, did not have a statistically significant influence on the surface finish because this effect was not included in the outcome of the last iteration for the ANOVA summarized in Table 8.

**Figure 6:**
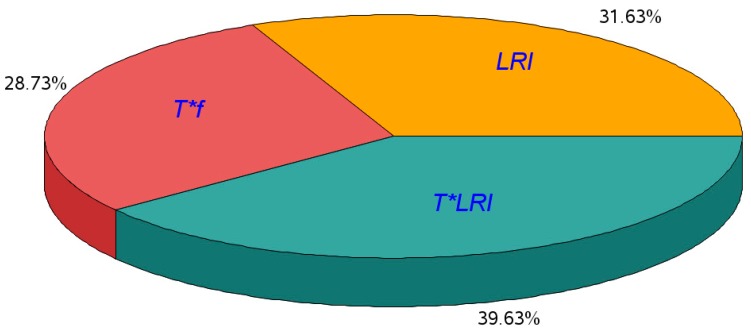
Distribution of the percentage of variability attributed to each effect over the variability explained by the ANOVA model.

Considering the variability in the surface roughness of the magnesium–titanium–magnesium drilling stacks explained by the statistically significant effects obtained from the ANOVA, the percentage of variability attributed to each effect is shown in the pictogram in Figure 6. The contribution of each effect was obtained as the ratio of the sum of squares of the effect to the sum of squares due to the model. For example, the percentage of variability attributed to the T*f effect is the ratio of 2.206 to 7.673 (%). That is, 39.63% of the variability is due to the T*LRI effect, 31.63% of the variability is due to the *LRI* effect, and the remaining 28.73% of the variability is due to the T*f effect.

**Figure 7:**
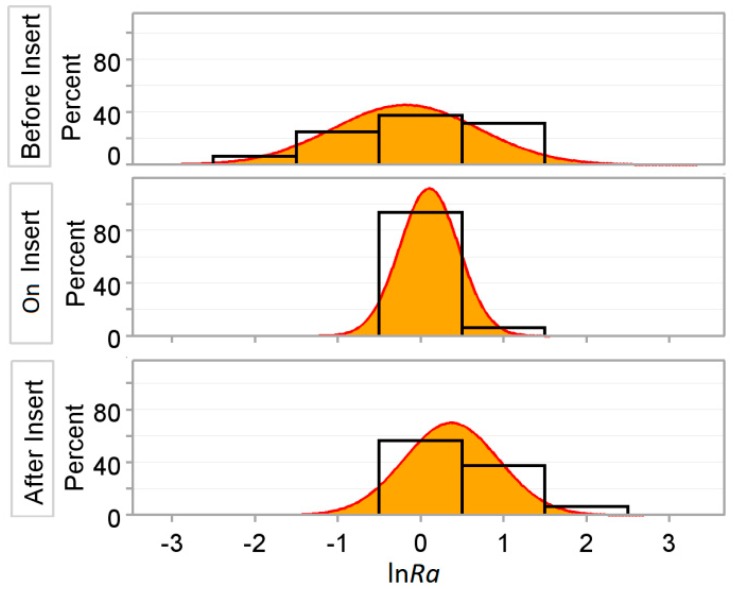
Distribution of the Naperian logarithm of the surface roughness (ln*Ra*) for each of the three levels of measurement location on the insert (*LRI*).

The distribution of the Naperian logarithm of the surface roughness of the drilling process (ln*Ra*) with respect to the three levels of the measurement location on the insert (*LRI)* is depicted in Figure 7. As shown in the figure, small differences existed between ln*Ra* before and after the insert. The dispersion is observably greater for small values before the insert. 

**Figure 8:**
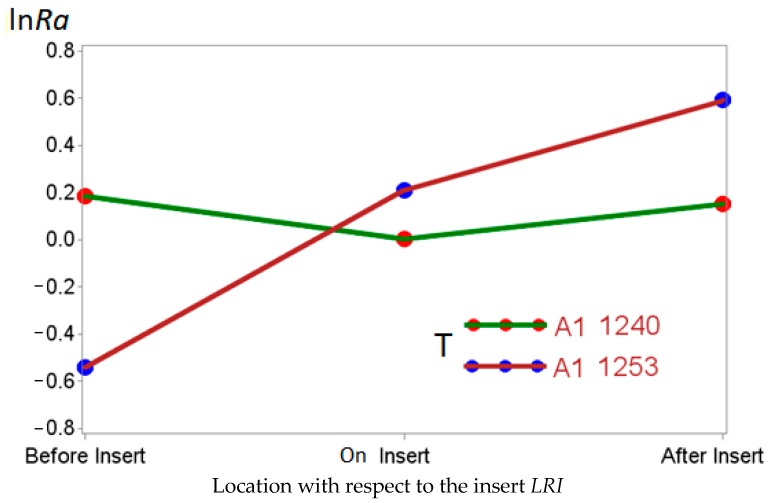
ln*Ra* interaction between type of tool and the location with respect to the insert T*LRI.

On the other hand, when assessing statistically significant interactions, considering the behavior of the interaction between type of tool and the location with respect to the insert T*LRI, the A1 1253 tool produced better results in terms of roughness before the insert and worse after the insert. This did not occur with the A1 1240 tool, which had a few differences. This behavior is illustrated in Figure 8.

**Figure 9:**
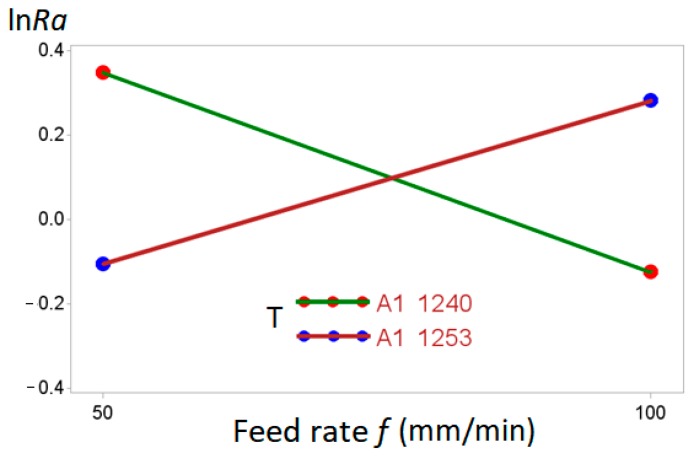
ln*Ra* interaction between type of tool and feed rate T*f.

With respect to the interaction between type of tool and feed rate T*f (Figure 9), an increase in the advance of *f* generated a reduction in the roughness for the A1 1240 tool, whereas for the A1 1253 tool, the opposite occurred. The figure clearly demonstrates the T*f interaction.

The variability in the logarithm of the surface roughness ln*Ra* of dry drilling magnesium–titanium–magnesium stacks was modelled from the ANOVA using Equation (1). In this equation, *µ* is a constant term to adjust the mean; *α_i_, βα_ji_*, and *βγ_jk_* represent the effects of the levels of the location with respect to the insert, the interaction of the type of tool with the measurement location with respect to the insert, and the interaction of the type of tool with the feed rate, respectively; and *ε_ijk_* is the error term.

ln*Ra_ijk_* = *µ + α_i_ + βα_ji_ + βγ_jk_ + ε_ijk_* (1)

The estimations of the parameters of the model in Equation (1) are listed in the third column of Table 9. The table also includes an indicator of the parameter estimation errors (the standard deviation) in the fourth column. The fifth column collects the *t*-statistic values, and the sixth column provides the statistical significances of the parameter estimations.

**Figure 10:**
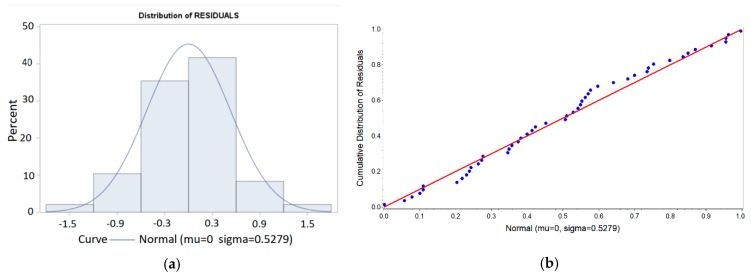
Checking the hypothesis of normality for the residuals: (**a**) histogram of residuals and (**b**) Probability–Probability plot for residuals.

The parameter estimations included in Table 9 allowed us to obtain the residuals of the model in Equation (2), which provides the differences between the observed values and the estimated values of ln*Ra*. Analyzing these residuals, the model hypotheses were checked. Figure 10 shows that the residuals follow a normal law, so the hypothesis is supported. This hypothesis can be contrasted by different tests, such as normality on residuals, as shown in Table 10.

**Figure 11:**
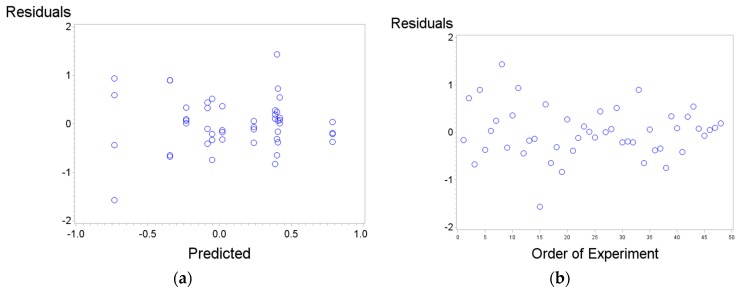
(**a**) Checking the homoscedasticity hypothesis for residuals; (**b**) Checking the nonexistence of special patterns on residuals.

Figure 11a shows that the residuals satisfy the homoscedasticity hypothesis and Figure 11b shows that the existence of nonrandomized patterns in the residuals was not observed.

Once the hypotheses of the model in Equation (1) were checked, we confirmed that the variability in the surface roughness *Ra* of dry drilling stacks composed of magnesium alloy UNS M11917 and titanium alloy UNS R56400 could be statistically modelled by Equation (2).

*Ra_ijk_* = exp (*µ + α_i_ + βα_ji_ + βγ_jk_ + ε_ijk_*) (2)

From modelling the surface roughness *Ra* described in Equation (2), and considering the parameter estimations in Table 9, the predicted values for surface roughness of the drilling machining were computed for the various combinations of the levels of the statistically significant effects in the surface finish of the dry drilling stacks. The values of the predicted roughness of these combinations are listed in increasing order in Table 11. The second predicted roughness, denoted with an asterisk (*) and included in the last column of the table, contains the predicted *Ra* values using only the parameters with a statistical significance at the *p* < 0.05 level. Considering this second roughness prediction (*), the combinations of the levels of statistically significant effects were classified into four roughness classes, as shown in the last column of Table 11.

Focusing on this classification, the best combinations of the levels of parameters that achieved a lower predicted roughness value (a better surface finish) during the dry drilling process on a stack composed of magnesium alloy UNS M11917 and titanium alloy UNS R56400 included the A1 1240 cutting tool with a feed rate of 100 mm/min. Appropriate levels of surface finish should be achieved by these drilling conditions: a predicted roughness of 0.92 µm on the insert and after the insert (in the second class), and a predicted roughness of 0.95 µm before the insert (in the third class). A mean roughness *Ra* under 1 µm could be achieved in all the stack superficial areas.

These types of light alloys are usually employed in the aeronautical industry and in this industrial sector, the values for the surface roughness specifications of *Ra* usually lie between 0.8 μm and 1.6 μm [91]. As such, roughness values *Ra* under 1 µm clearly satisfy the surface finish requirements. Notably, the quality improvement in the surface finish was achieved with higher feed rates, which promoted a decrease in machining time and, consequently, a decrease in costs, enabling the efficient optimization of the surface quality.

### 4.3. Technological Point of View

To apply the results obtained in this study, analyzing them from a technological point of view was necessary. Thus, as most components in the aeronautical and aerospace sectors are complex and have strict dimensional and surface quality requirements (within the range of 0.8 μ < *Ra* < 1.6 μm [94]), their manufacturing is usually expensive and pieces are not stocked for repair or maintenance purposes. Therefore, parts have to be repaired or maintained as soon as possible to restore the plane or the aircraft to its functional conditions.

In this study, the best results were obtained for the following combinations of cutting parameters: *f* = 100 mm/min, *V* = 25, and *T* = A1 1240 m/min; and *f* = 50 mm/min, *V* = 25, and *T* = A1 1253 m/min. The surface roughness achieved in the holes of the different materials and locations in the stack were all within the range usually required in the aeronautical industry [94]. Between the two combinations, the first was better due to the feed rate value being higher so the repair operation can be finished in a shorter time, thereby reducing costs associated with the operation. Additionally, these real results are in accordance with the results of the ranking of the cutting condition combinations based on the estimated values of *Ra*.

Performing all tests under dry conditions is important not only from an economic point of view but an environmental viewpoint as well. Not using any additional lubricant or coolant provides cost savings and allows for more sustainable repair and maintenance operations.

## 5. Conclusions

In this paper, we examined a drilling process on a stack formed by two UNS M11917 magnesium alloy bases and one UNS R56400 titanium alloy insert in an experimental study. The interaction between the type of tool and the measurement location on the insert influences the inner surface of the holes. The best type of tool for the drilling repair operations was determined to be the A1 1240, which was especially efficient for higher values of feed rate (100 mm/min) and cutting speed (25 m/min). The surface roughness obtained in the inner of the holes was independent of material and location considered, and the values fell within the usual acceptable range in the aeronautical sector. The surface roughness increased as the tool advanced through the stack, especially after the insert. However, the differences observed along each component in the stack, at both the beginning and the end of the component, were not statistically significant. The repair operations performed with drilling can be sustainably completed, as was proven in this study, which was completed under dry conditions.

## Figures and Tables

**Table 1:** Factors and levels.

Factor	Level
Feed rate, *f* (mm/min)	*f1*, *f2*
Cutting speed, *V* (m/min)	*V1*, *V2*
Tool coating type, *T*	*T1*, *T2*
Location with respect to the specimen, *LRS*	*LRS1*, *LRS2*
Location with respect to the insert, *LRI*	*LRI1*, *LRI2*, *LRI3*

**Table 2:** Experimental design: product of a full factorial 2^3^ and a block of two factors 3 × 2.

*T*	*V* (m/min)	*f* (mm/min)	*LRI*	*LRS*	
*T1*	*V1*	*f2*	*LRI2*	*LRS1*	*LRS2*
*T1*	*V1*	*f2*	*LRI1*	*LRS1*	*LRS2*
*T1*	*V1*	*f2*	*LRI3*	*LRS1*	*LRS2*
*T1*	*V1*	*f1*	*LRI3*	*LRS1*	*LRS2*
*T1*	*V1*	*f1*	*LRI2*	*LRS1*	*LRS2*
*T1*	*V1*	*f1*	*LRI1*	*LRS1*	*LRS2*
*T1*	*V2*	*f1*	*LRI2*	*LRS1*	*LRS2*
*T1*	*V2*	*f1*	*LRI1*	*LRS1*	*LRS2*
*T1*	*V2*	*f1*	*LRI3*	*LRS1*	*LRS2*
*T2*	*V1*	*f1*	*LRI3*	*LRS1*	*LRS2*
*T2*	*V1*	*f1*	*LRI2*	*LRS1*	*LRS2*
*T2*	*V1*	*f1*	*LRI1*	*LRS1*	*LRS2*
*T2*	*V2*	*f2*	*LRI3*	*LRS1*	*LRS2*
*T2*	*V2*	*f2*	*LRI2*	*LRS1*	*LRS2*
*T2*	*V2*	*f2*	*LRI1*	*LRS1*	*LRS2*
*T1*	*V2*	*f2*	*LRI3*	*LRS1*	*LRS2*
*T1*	*V2*	*f2*	*LRI1*	*LRS1*	*LRS2*
*T1*	*V2*	*f2*	*LRI2*	*LRS1*	*LRS2*
*T2*	*V1*	*f2*	*LRI1*	*LRS1*	*LRS2*
*T2*	*V1*	*f2*	*LRI2*	*LRS1*	*LRS2*
*T2*	*V1*	*f2*	*LRI3*	*LRS1*	*LRS2*
*T2*	*V2*	*f1*	*LRI1*	*LRS1*	*LRS2*
*T2*	*V2*	*f1*	*LRI2*	*LRS1*	*LRS2*
*T2*	*V2*	*f1*	*LRI3*	*LRS1*	*LRS2*

**Table 3:** Chemical compositions of materials used for manufacturing specimens.

UNS M11917 (AZ91D)	UNS R56400 (Ti-6Al-4V)
Al 8.30–9.70%	Al 5.5–6.75%
Cu ≤ 0.03%	C ≤ 0.08%
Fe ≤ 0.005%	H ≤ 0.015%
Mg 90%	Fe ≤ 0.4%
Mn ≥ 0.13%	N ≤ 0.03%
Ni ≤ 0.002%	O ≤ 0.2%
Si ≤ 0.1%	Ti 87.725–91%
Zn 0.35–1%	Zn 3.5–4.5%

**Table 4:** Factor and level values.

Factor	Level Value
Feed rate, *f*, (mm/min)	50/100
Cutting speed, *V*, (m/min)	20/25
Type of tool, *T*	A1 1253/A1 1240
Location with respect to the specimen, *LRS*	Beginning of the specimen, end of the specimen
Location with respect to the insert, *LRI*	Before the insert, on the insert, after the insert

**Table 5:** Roughness average *Ra* (μm) obtained during the experiments.

*T*	*V* (m/mnin)	*f* (mm/min)	*LRI*	*Ra* (μm)	
				*LRS1*	*LRS2*
A11253	20	100	*LRI2*	1.28	3.09
A11253	20	100	*LRI1*	0.36	1.73
A11253	20	100	*LRI3*	1.52	2.28
A11253	20	50	*LRI3*	1.91	6.28
A11253	20	50	*LRI2*	0.74	1.46
A11253	20	50	*LRI1*	1.23	0.31
A11253	25	50	*LRI2*	0.86	0.89
A11253	25	50	*LRI1*	0.10	0.87
A11253	25	50	*LRI3*	0.78	1.09
A11240	20	50	*LRI3*	0.64	1.94
A11240	20	50	*LRI2*	0.86	1.13
A11240	20	50	*LRI1*	1.73	1.54
A11240	25	100	*LRI3*	0.83	1.43
A11240	25	100	*LRI2*	0.80	0.85
A11240	25	100	*LRI1*	1.59	0.77
A11253	25	100	*LRI3*	1.81	1.79
A11253	25	100	*LRI1*	1.73	0.37
A11253	25	100	*LRI2*	1.60	1.03
A11240	20	100	*LRI1*	0.68	0.45
A11240	20	100	*LRI2*	1.11	0.87
A11240	20	100	*LRI3*	0.61	1.28
A11240	25	50	*LRI1*	2.62	1.65
A11240	25	50	*LRI2*	1.19	1.34
A11240	25	50	*LRI3*	1.63	1.78

**Table 6:** *Ra* (μm) mean values in the three measured zones versus the feed rate, *f*, and the cutting speed, *V*.

*Ra* (μm)	*f* (mm/min)		*V* (m/min)	
	50	100	20	25
*Ra*_LRI1_	1.26	0.96	1.00	1.21
*Ra*_LRI2_	1.06	1.33	1.32	1.08
*Ra*_LRI3_	2.01	1.44	2.06	1.39

**Table 7:** Outcome of the first iteration for the ANOVA over *Ra* Naperian logarithm.

Effect	DF	Sum of Squares	Mean Square	*F*-Value	Pr > *F*
*LRI*	2	2.426	1.213	3.77	0.036
*T*	1	0.007	0.007	0.02	0.823
T*LRI	2	3.041	1.521	4.73	0.017
*f*	1	0.021	0.021	0.06	0.802
LRI*f	2	0.225	0.112	0.35	0.708
T*f	1	2.206	2.206	6.86	0.014
*LRS*	1	0.492	0.492	1.53	0.227
LRI*LRS	2	0.886	0.443	1.38	0.270
T*LRS	1	0.177	0.177	0.55	0.465
f*LRS	1	0.214	0.214	0.67	0.422
*V*	1	0.092	0.092	0.29	0.597
LRI*V	2	0.122	0.061	0.19	0.828
T*V	1	1.565	1.565	4.86	0.036
f*V	1	0.211	0.211	0.65	0.425
V*LRS	1	0.429	0.429	1.33	0.259
Error	27	8.686	0.322		
Total	47	20.800			

DF: Degrees of Freedom.

**Table 8:** Outcome of the last iteration for the ANOVA over *Ra* Naperian logarithm.

Effect	DF	Sum of Squares	Mean Square	*F*-Value	Pr > *F*
T*f	1	2.206	2.206	7.06	0.011
*LRI*	2	2.426	1.213	3.88	0.028
T*LRI	2	3.041	1.521	4.87	0.013
Error	42	13.127	0.313		
Total	47	20.800			

DF: Degrees of Freedom.

**Table 9:** Parameter estimations of the model in Equation (1).

Parameter	Designation	Estimation	Standard Error	*t*-Value	Pr > |*t*|
Intercept	*µ*	0.7862	0.2336	3.37	0.0017
A1 1253*50 (mm/min)	*βγ*_11_	−0.3873	0.2336	−1.66	0.1052
A1 1253*100 (mm/min)	*βγ*_12_	0	.	.	.
A1 1240*50 (mm/min)	*βγ*_21_	−0.4004	0.3304	−1.21	0.2326
A1 1240*100 (mm/min)	*βγ*_22_	−0.8706	0.3304	−2.64	0.0119
Before the insert	*α*_1_	−1.1333	0.2861	−3.96	0.0003
On the insert	*α*_2_	−0.3820	0.2861	−1.34	0.1894
After the insert	*α*_3_	0	.	.	.
A1 1253*Before the insert	*βα*_11_	0	.	.	.
A1 1253*On the insert	*βα*_12_	0	.	.	.
A1 1253*After the insert	*βα*_13_	0	.	.	.
A1 1240*Before the insert	*βα*_21_	1.1654	0.4046	2.88	0.0064
A1 1240*On the insert	*βα*_22_	0.2335	0.4046	0.58	0.5671
A1 1240*After the insert	*βα*_23_	0	.	.	.

**Table 10:** Tests for normality on residuals.

Test for Normality	Statistic		*p*-Value	
Shapiro–Wilk	W	0.977919	Pr < W	0.4952
Kolmogorov–Smirnov	D	0.0900	Pr > D	>0.150

**Table 11:** Predicted roughness of the level combinations of significant effects on dry drilling.

*LRI*	*T*	*f* (mm/min)	ln*Ra* Predicted *α_i_ + βα_ji_ + βγ_jk_*	*Ra* Predicted (µm) exp (*α_i_ + βα_ji_ + βγ_jk_*)	*Ra* Predicted * (µm) exp (α_i_^*^ + βα_ji_^*^ + βγ_jk_^*^)
Before insert	A1 1253	50	−0.734	0.48	0.71 (Class I)
Before insert	A1 1253	100	−0.347	0.71	
On insert	A1 1240	100	−0.233	0.79	0.92 (Class II)
After insert	A1 1240	100	−0.084	0.92	
Before insert	A1 1240	100	−0.052	0.95	0.95 (Class III)
On insert	A1 1253	50	0.017	1.02	2.20 (Class IV)
On insert	A1 1240	50	0.237	1.27	
After insert	A1 1240	50	0.386	1.47	
After insert	A1 1253	50	0.399	1.49	
On insert	A1 1253	100	0.404	1.50	
Before insert	A1 1240	50	0.418	1.52	
After insert	A1 1253	100	0.786	2.20	
